# Supplementary material for: Room temperature and low-field resonant enhancement of spin Seebeck effect in partially compensated magnets
Source: Nat Commun. 2019 Nov 14;10:5162. doi: 10.1038/s41467-019-13121-5 (PMC6856150; doi:10.1038/s41467-019-13121-5)
Supplement: Supplementary file 1 — Supplementary Information [file 41467_2019_13121_MOESM1_ESM.pdf]

Room temperature and low-field resonant enhancement of spin Seebeck effect in partially compensated magnets

R. Ramos et al.*

SUPPLEMENTARY NOTE 1: STRUCTURAL CHARACTERIZATION

The structural quality of the $Lu_2BiFe_4GaO_{12}$ (BiGa:LuIG) film used in the present study was confirmed by X-ray diffraction (XRD) and high-resolution transmission electron microscopy (TEM) measurements. The structural characterization of the LPE-grown YIG used for comparison has been described elsewhere[1]. Supplementary Figure 1a shows the XRD results of the $2\theta - \omega$ scan measured in the symmetric configuration around the (004) peak of the $Gd_3Ga_5O_{12}$ (GGG) substrate at $2\theta \sim 28.83$ °. It can be clearly seen that the BiGa:LuIG film peak is at slightly larger angles than that of the GGG, indicating the lower lattice parameter of the film, as expected: $a_{\mathrm{GGG}} = 1.2383$ nm, $a_{\mathrm{LuIG}} = 1.2284$ nm [red line shows expected 2θ value of (004) peak for bulk $Lu_3Fe_5O_{12}$ (LuIG)]. The Bi-substitution increases the lattice parameter of LuIG to a value closer to that of GGG[2] and due to the good lattice matching with the GGG substrate we cannot fully resolve the thin film peak. To further check the structural quality of the sample, we also performed symmetric and antisymmetric reciprocal space mapping (RSM) around the (004) and $(\bar{1}\bar{1}6)$ peaks of the GGG substrate, respectively. The result of the RSM around the (004) peak is fully consistent with the $2\theta - \omega$ scan, having a brighter intensity for values of Miller index l larger than that of the GGG peak (Supplementary Figure 1b). In the case of the RSM around the GGG $(\bar{1}\bar{1}6)$ peak, the alignment of the film reflection with the same value of h as that of the GGG, indicates that film and substrate have the same in-plane lattice parameter and confirms the pseudomorphic growth of BiGa:LuIG on GGG (Supplementary Figure 1c).

To further investigate the structural quality of the GGG/BiGa:LuIG/Pt bilayer, we performed transmission electron microscopy (TEM) and bright field scanning TEM (BF-STEM) (Supplementary Figures 1e to 1j). In supplementary Figures 1e (1f), the TEM image of the bilayer in the region close to the BiGa:LuIG/Pt (GGG/BiGa:LuIG) interface shows that the sample is homogenous having sharp interfaces and no interdifussion.

We have also performed high resolution TEM (HR-TEM) (Supplementary Figures 1g, 1h) and HR-BF-STEM (Supplementary Figures 1i, 1j) measurements. If we now take a closer look at the GGG/BiGa:LuIG interface, shown in Supplementary Figures 1h and 1j, we can see that the BiGa:LuIG grows epitaxially on GGG with a sharp, smooth interface, perfect lattice matching and absence of defects. In fact, the film and substrate are nearly indistinguishable at the interface. This is fully consistent with the XRD analysis.

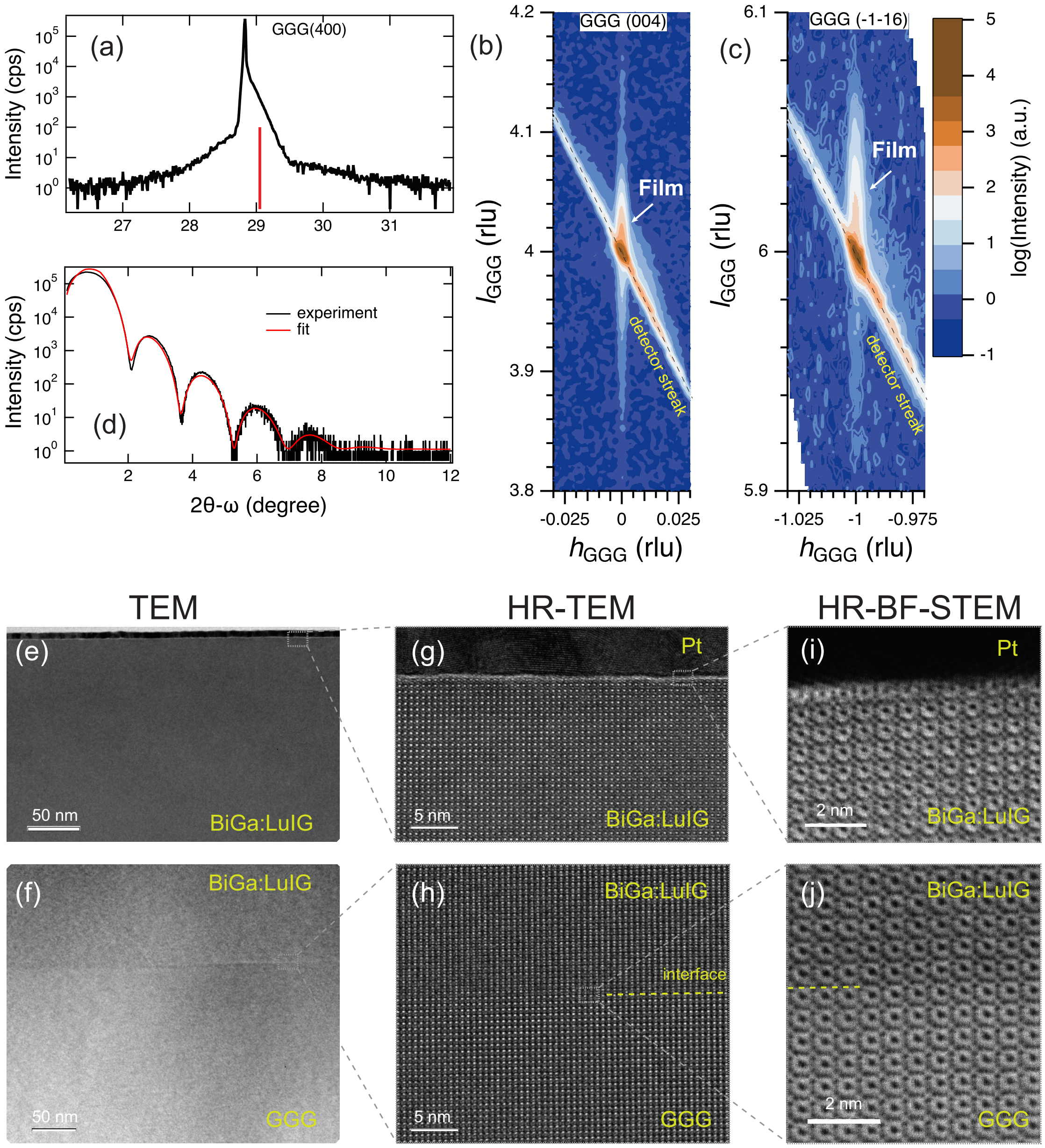

Supplementary Figure 1. **a** XRD of GGG//BiGa:LuIG measured around the vicinity of the (004) peak of GGG (red line shows expected peak value for bulk undoped LuIG). **b-c** Symmetric and antisymmetric RSM of GGG//BiGa:LuIG around the GGG (004) **b** and GGG($\bar{1}\bar{1}6$) **c** peaks. The position of the film peak is nearly indistinguishable from that of substrate peak due to the close lattice matching. The dotted line is an instrumental artifact (detector streak) not related to the properties of the sample. **d** XRR measurement in specular reflection, representing the properties of the BiGa:LuIG/Pt interface, the period of the oscillations is inversely proportional to the Pt thickness. The red line shows the result of the fitting performed using the DIFFRAC^{plus} LEPTOS 7 software by BRUKER, where we obtained a platinum thickness $t_{\mathrm{Pt}} = 5.1 \pm 0.4$ nm. **e-f** Transmission electron microscopy (TEM) images of the BiGa:LuIG/Pt **e** and GGG/BiGa:LuIG **f** interfaces. High resolution (HR-TEM) **g-h** and HR bright field scanning TEM (HR-BF-STEM) **i-j** images confirming the quality of the GGG/BiGa:LuIG **h-j** and BiGa:LuIG/Pt **g-i** interfaces.

Now, if we look at the BiGa:LuIG/Pt interface (see Supplementary Figures 1g and 1i for the HR-TEM and HR-BF-STEM, respectively), we can see a clean and sharp interface. By inspecting the HR-BF-STEM (Supplementary Figure 1i), we can confirm that the interface is atomically sharp, having a roughness even smaller than that obtained from the fitting of the results of X-ray reflectivity (XRR) measurement (Supplementary Figure 1d). The condition of the BiGa:LuIG/Pt interface in the present experiment is consistent with that previously reported by Qiu et al.[1] in YIG/Pt for Pt deposited at room temperature directly on the YIG after mechanically polish of the surface and without prior heat treatment (as it was performed in the present study and the studies on SSE suppression and magnon-polaron SSE in YIG/Pt by Kikkawa et al.[3,4]). Moreover, the atomic sharpness of the interface allows us to rule out effects from surface roughness at the BiGa:LuIG interface[5]

SUPPLEMENTARY NOTE 2: MAGNETIC CHARACTERIZATION

A. Temperature dependence of the magnetization

We measured the magnetic field dependence of the magnetization at different temperatures using a vibrating sample magnetometer (VSM) in a physical property measurements system (PPMS) of Quantum Design, Inc. (Supplementary Figures 2a to 2d). The temperature dependence of the saturation magnetization, M_{S} was then obtained from the value measured at 30 mT for each temperature. Supplementary Figure 2e shows the comparison between the M_{S} for BiGa:LuIG and YIG measured at different temperatures (the temperature dependence of the M_{S} of BiGa:LuIG is also shown in Fig. 4b of the main text), it can be clearly seen that the Ga-induced magnetic compensation results in a strong reduction of the magnetization and magnetic ordering temperature (T_{c}) of BiGa:LuIG compared to YIG, as expected[6]. We should note that the value of M_{S} and T_{c} for undoped LuIG is almost the same as that of YIG[7,8], this is essentially due to the fact that neither Lu or Y posses magnetic moment and in both systems the ferrimagnetic order arises from the imbalance between Fe occupying the tetrahedral (d) and octahedral (a) sites with a ratio of 3:2 (d:a), resulting in a non-zero magnetic moment.

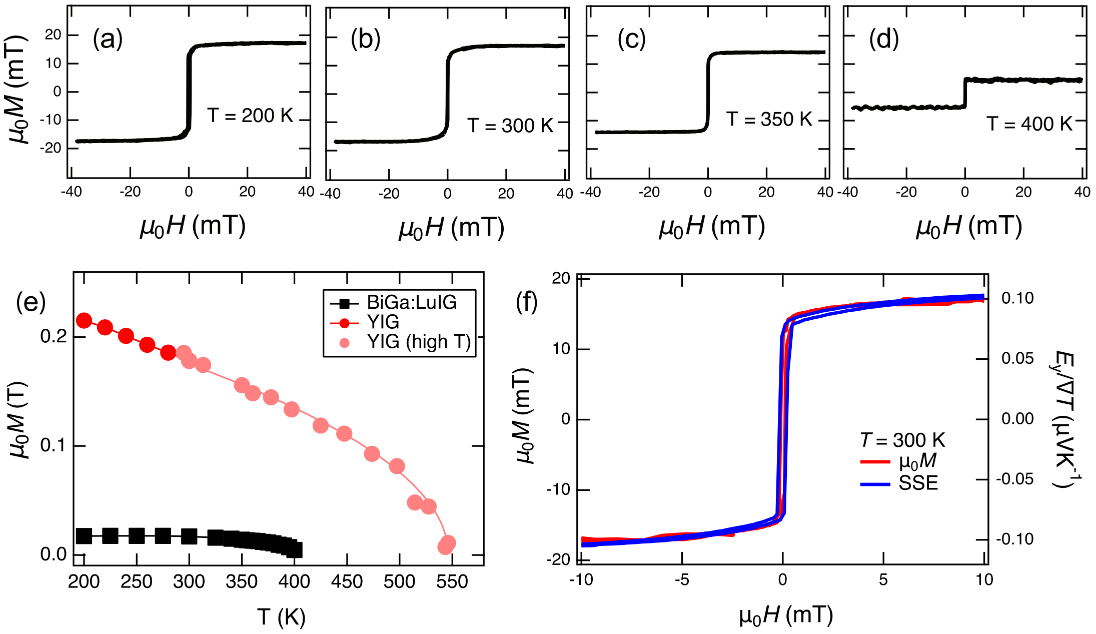

Supplementary Figure 2. Magnetic field dependence of the magnetization of BiGa:LuIG measured at **a** 200 K, **b** 300 K, **c** 350 K, **d** 400 K. **e** Temperature dependence of the saturation magnetization M_S of BiGa:LuIG film (solid black squares), obtained from the magnetization hysteresis loops at each temperature and its comparison to the magnetization of YIG (red symbols) (data for YIG below room temperature (dark red) and above room temperature (light red) were extracted from previous studies on YIG film grown by liquid phase epitaxy in T. Kikkawa et al.[3] and K. Uchida et al.[9], respectively). **f** Detail of the low magnetic field dependence of the magnetization (left axis) and SSE (right axis) measured at 300 K.

B. Comparison between magnetization and SSE at low magnetic fields

It has been previously shown that the competition between bulk and surface anisotropies in YIG can result in pronounced differences in the magnetic field dependence behavior of the magnetization and the SSE at low fields.[5,10–12] This effect is quite pronounced for YIG slabs and gradually decreases as the YIG thickness is reduced, being absent for films with thickness lower than 5 μm[10]. Here, in order to confirm the absence of this effect in the BiGa:LuIG(4μm) film, we have performed precise measurements of the SSE and magnetization at low magnetic fields. Supplementary Figure 2f shows the comparison between the magnetic field response of the magnetization and SSE measured at room temperature, we can clearly see that the magnetic field dependence of the SSE and magnetization closely follow each other upon approaching the saturation magnetization, therefore ruling out differences in the magnetic field response due to the competition between surface and bulk anisotropies in the SSE at low magnetic fields.

SUPPLEMENTARY NOTE 3: MAGNON LIFETIME OF BIGA:LUIG

We have performed time-resolved Brillouin light scattering (BLS) measurements to estimate the magnon lifetime in BiGa:LuIG system. Time-resolved BLS was performed in the back-scattering configuration with a microwave input modulated to square wave with the duration of 150 ns. The sample was placed on a microstrip antenna which excites an uniform precession ($k = 0$ magnons) in the film. The scattered light is collected with an objective lens and analyzed by a Fabry-Perot interferometer. The time resolution of this measurement is determined by the finesse of the Fabry-Perot interferometer and is 1 ns[13]. Supplementary Figure 3 shows a schematic and the obtained result for an applied microwave excitation of 3.24 GHz. By fitting the tail of the curve to an exponential decay[14] we can estimate the magnon lifetime of the system studied here, obtaining $\tau_{\mathrm{m}}(\mathrm{BiGa{:}LuIG}) = 12.63 \pm 1.21$ ns, which is strongly decreased compared to the case of YIG system [$\tau_{\mathrm{m}}(\mathrm{YIG}) =$ 50 to 75 ns][14,15]. Therefore supporting the interpretation that the larger enhancement of the magnitude of the magnon-polaron SSE in BiGa:LuIG with respect to YIG is due to the decrease of the magnon lifetime in BiGa:LuIG as a result of doping.

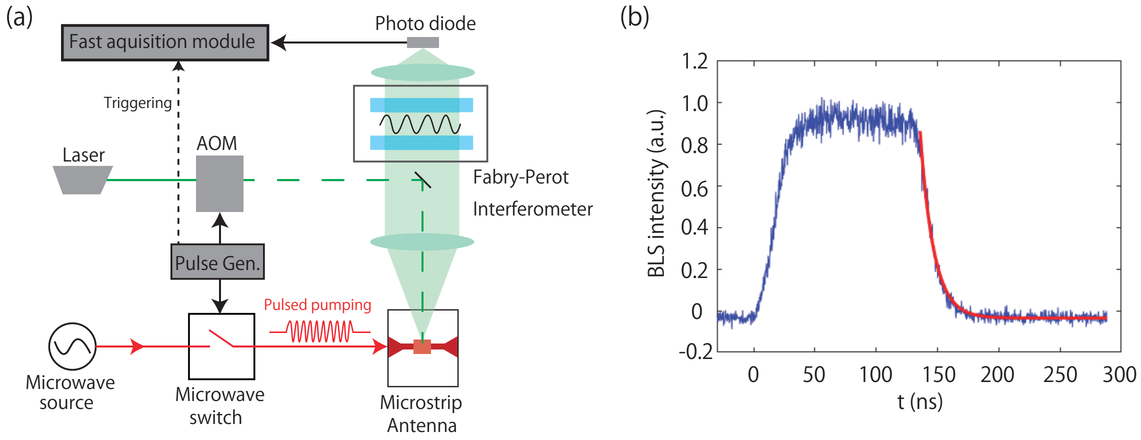

Supplementary Figure 3. **a** Schematic of the experimental setup for time-resolved BLS measurements. **b** Representative example of the obtained data with the corresponding fitting to estimate the magnon lifetime.

SUPPLEMENTARY NOTE 4: DERIVATION OF THE MAGNON DISPERSION FOR A TWO-SUBLATTICE FERRIMAGNETIC SYSTEM WITH DIFFERENT SITE OCCUPATION

Let us write the Hamiltonian for a ferrimagnet as a function of the number of ions occupying the different magnetic sublattices of the system, then we can obtain an expression dependent on the level of substitution by non-magnetic ions. We start with the Hamiltonian which considers the exchange interactions between neighboring spins, and of the individual spins with an external magnetic field (Zeeman interaction). We neglect the effect of dipolar interaction or magnetic anisotropy to simplify the treatment. Here, since $|J_{\mathrm{ad}}| >> |J_{\mathrm{aa}}|, |J_{\mathrm{dd}}|$[16,17] only inter-site exchange interaction term is considered. Then, we can express the Hamiltonian:

$$
\begin{aligned}
H = & \frac{J_{\mathrm{ad}}}{\hbar^2}\sum_{i,\delta}^{N_{\mathrm{d}}} \mathbf{S}_{\mathrm{d},i}\mathbf{S}_{\mathrm{a},i+\delta} - \frac{g\mu_{\mathrm{B}}B_0}{\hbar}\sum_{i}^{N_{\mathrm{d}}} S^z_{\mathrm{d},i} \\
& + \frac{J_{\mathrm{ad}}}{\hbar^2}\sum_{j,\delta}^{N_{\mathrm{a}}} \mathbf{S}_{\mathrm{d},j+\delta}\mathbf{S}_{\mathrm{a},j} - \frac{g\mu_{\mathrm{B}}B_0}{\hbar}\sum_{j}^{N_{\mathrm{a}}} S^z_{\mathrm{a},j},
\end{aligned} \tag{1}
$$

here we use a positive value for the nearest-neighbour inter-sublattice exchange constant J_{ad}, δ represents a vector connecting the nearest-neighbour a-d sites, $\mathbf{S}_{\mathrm{x}}$ (x = a, d) is the spin operator for a, d sites, g is the spectroscopic splitting factor, μ_{B} the Bohr magneton, $\hbar$ the reduced Planck constant and B_0 the external magnetic field. We separated the Hamiltonian $H = H_{\mathrm{d}} + H_{\mathrm{a}}$ into the contributions from the tetrahedral (H_{d}) and octahedral (H_{a}) sites, with magnetic ion numbers per unit volume N_{d} and N_{a}, respectively. The spin operators $\mathbf{S}_{\mathrm{a},i}$, $\mathbf{S}_{\mathrm{d},i}$ can be expressed in terms of the spin raising and lowering operators, defined as: $S^+_{\mathrm{m},i} = S^x_{\mathrm{m},i} + iS^y_{\mathrm{m},i}$ and $S^-_{\mathrm{m},i} = S^x_{\mathrm{m},i} - iS^y_{\mathrm{m},i}$, with m = a, d. Then we can consider the Holstein-Primakoff approximation[18,19], which express the spin creation and annihilation operators in

terms of boson operators that create or destroy magnons, and are given by:

$$\begin{aligned}
S^{+}_{\mathrm{d},i} &= (2S_{\mathrm{d}})^{1/2}\hbar\left(1-\frac{a_i^{\dagger}a_i}{2S_{\mathrm{d}}}\right)^{1/2}a_i \simeq (2S_{\mathrm{d}})^{1/2}\hbar a_i\\
S^{-}_{\mathrm{d},i} &= (2S_{\mathrm{d}})^{1/2}\hbar a_i^{\dagger}\left(1-\frac{a_i^{\dagger}a_i}{2S_{\mathrm{d}}}\right)^{1/2} \simeq (2S_{\mathrm{d}})^{1/2}\hbar a_i^{+}\\
S^{+}_{\mathrm{a},j} &= (2S_{\mathrm{a}})^{1/2}\hbar b_j^{\dagger}\left(1-\frac{b_j^{\dagger}b_j}{2S_{\mathrm{a}}}\right)^{1/2} \simeq (2S_{\mathrm{a}})^{1/2}\hbar b_j^{\dagger}\\
S^{-}_{\mathrm{a},j} &= (2S_{\mathrm{a}})^{1/2}\hbar\left(1-\frac{b_i^{\dagger}b_j}{2S_{\mathrm{a}}}\right)^{1/2}b_j \simeq (2S_{\mathrm{a}})^{1/2}\hbar b_j\\
S^{z}_{\mathrm{d},i} &= (S_{\mathrm{d}}-n_i)\hbar\\
S^{z}_{\mathrm{a},j} &= (-S_{\mathrm{a}}+n_j)\hbar\\
a_i^{\dagger}a_i &= n_i\\
b_j^{\dagger}b_j &= n_j,
\end{aligned} \tag{2}$$

where $S_{\mathrm{d}}(S_{\mathrm{a}})$ is spin at the tetrahedral (octahedral) site, $a_i^{\dagger}$ ($b_i^{\dagger}$) and a_i (b_i) are the magnon creation and annihilation operators for the d (a) site, respectively, which satisfy the boson commutation rules $[a_i, a_l^{\dagger}] = [b_i, b_l^{\dagger}] = \delta_{i,l}$ and $[a_i, a_j] = [b_i, b_j] = 0$. Using the above transformations, the terms of the Hamiltonian of Eq. 1: $H = H_{\mathrm{d}} + H_{\mathrm{a}}$ can be expressed as (up to second order in the magnon creation/annihilation operators):

$$H_{\mathrm{d}} = J_{\mathrm{ad}}\sum_{i,\delta}^{N_{\mathrm{d}}}\left[(S_{\mathrm{a}}S_{\mathrm{d}})^{1/2}(a_i^{\dagger}b_{i+\delta}^{\dagger}+a_ib_{i+\delta}) - S_{\mathrm{a}}S_{\mathrm{d}} + S_{\mathrm{d}}b_{i+\delta}^{\dagger}b_{i+\delta} + S_{\mathrm{a}}a_i^{\dagger}a_i\right] - g\mu_{\mathrm{B}}B_0\sum_{i}^{N_{\mathrm{d}}}(S_{\mathrm{d}}-a_i^{\dagger}a_i), \tag{3}$$

and

$$H_{\mathrm{a}} = J_{\mathrm{ad}}\sum_{j,\delta}^{N_{\mathrm{a}}}\left[(S_{\mathrm{a}}S_{\mathrm{d}})^{1/2}(a_{j+\delta}^{\dagger}b_j^{\dagger}+a_{j+\delta}b_j) - S_{\mathrm{a}}S_{\mathrm{d}} + S_{\mathrm{d}}b_j^{\dagger}b_j + S_{\mathrm{a}}a_{j+\delta}^{\dagger}a_{j+\delta}\right] - g\mu_{\mathrm{B}}B_0\sum_{i}^{N_{\mathrm{a}}}(-S_{\mathrm{a}}+b_j^{\dagger}b_j), \tag{4}$$

then if we consider the Fourier transforms (and their inverse) for the magnon operators a_i and b_j: $a_j = N_{\mathrm{d}}^{-1/2}\sum_k e^{-i\mathbf{k}\cdot\mathbf{r}_j}a_{\mathbf{k}}$ ($a_j^{\dagger} = N_{\mathrm{d}}^{-1/2}\sum_k e^{i\mathbf{k}\cdot\mathbf{r}_j}a_{\mathbf{k}}^{\dagger}$) and $b_j = N_{\mathrm{a}}^{-1/2}\sum_k e^{i\mathbf{k}\cdot\mathbf{r}_j}b_{\mathbf{k}}$ ($b_j^{\dagger} = N_{\mathrm{a}}^{-1/2}\sum_k e^{-i\mathbf{k}\cdot\mathbf{r}_j}b_{\mathbf{k}}^{\dagger}$), and the definition of the delta Kronecker function: $\delta_{\mathbf{k},\mathbf{k}'} =$

$\frac{1}{N_{\rm a,d}}\sum_i^{N_{\rm a,d}} e^{i(\mathbf{k}-\mathbf{k}')\cdot\vec{r}_i}$, we obtain the expression below:

$$H_{\rm d} = J_{\rm ad}\left(\frac{S_{\rm a}S_{\rm d}}{N_{\rm a}N_{\rm d}}\right)^{1/2}\sum_{\boldsymbol{\delta}\mathbf{k}}\left[N_{\rm d}\left(e^{-i\mathbf{k}\cdot\boldsymbol{\delta}}a_{\mathbf{k}}^\dagger b_{\mathbf{k}}^\dagger + e^{i\mathbf{k}\cdot\boldsymbol{\delta}}a_{\mathbf{k}}b_{\mathbf{k}}\right) - N_{\rm d}S_{\rm a}S_{\rm d} + S_{\rm d}\frac{N_{\rm d}}{N_{\rm a}}b_{\mathbf{k}}^\dagger b_{\mathbf{k}} + S_{\rm a}a_{\mathbf{k}}^\dagger a_{\mathbf{k}}\right] - g\mu_{\rm B}B_0\left(N_{\rm d}S_{\rm d} - \sum_{\mathbf{k}} a_{\mathbf{k}}^\dagger a_{\mathbf{k}}\right), \tag{5}$$

and

$$H_{\rm a} = J_{\rm ad}\left(\frac{S_{\rm a}S_{\rm d}}{N_{\rm a}N_{\rm d}}\right)^{1/2}\sum_{\boldsymbol{\delta}\mathbf{k}}\left[N_{\rm a}\left(e^{i\mathbf{k}\cdot\boldsymbol{\delta}}a_{\mathbf{k}}^\dagger b_{\mathbf{k}}^\dagger + e^{-i\mathbf{k}\cdot\boldsymbol{\delta}}a_{\mathbf{k}}b_{\mathbf{k}}\right) - N_{\rm a}S_{\rm a}S_{\rm d} + S_{\rm d}b_{\mathbf{k}}^\dagger b_{\mathbf{k}} + S_{\rm a}\frac{N_{\rm a}}{N_{\rm d}}a_{\mathbf{k}}^\dagger a_{\mathbf{k}}\right] - g\mu_{\rm B}B_0\left(-N_{\rm a}S_{\rm a} + \sum_{\mathbf{k}} b_{\mathbf{k}}^\dagger b_{\mathbf{k}}\right), \tag{6}$$

which can be further simplified by defining $\gamma_k = \gamma_{-k} = 1/z_i\sum_\delta e^{ik\delta}$, with $z_i = z_{\rm ad}$ or $z_{\rm da}$ the number of nearest neighbors for octahedral and tetrahedral sites, respectively. Then the Hamiltonian of the ferrimagnet can be expressed as:

$$H = -g\mu_{\rm B}B_0(N_{\rm d}S_{\rm d} - N_{\rm a}S_{\rm a}) - J_{\rm ad}S_{\rm a}S_{\rm d}(N_{\rm d}z_{\rm da} + N_{\rm a}z_{\rm ad}) + \sum_k H_k, \tag{7}$$

where the first two terms in the right side describe the ground state energy[20] and the third term describes the magnon excitation and is given by:

$$\begin{aligned} H_k = {} & J_{\rm ad}\left[\frac{S_{\rm a}}{N_{\rm d}}(z_{\rm ad}N_{\rm a} + z_{\rm da}N_{\rm d}) + g\mu_{\rm B}B_0\right]a_k^\dagger a_k \\ & + J_{\rm ad}\left[\frac{S_{\rm d}}{N_{\rm a}}(z_{\rm ad}N_{\rm a} + z_{\rm da}N_{\rm d}) - g\mu_{\rm B}B_0\right]b_k^\dagger b_k \\ & + J_{\rm ad}\left(\frac{S_{\rm a}S_{\rm d}}{N_{\rm a}N_{\rm d}}\right)^{1/2}\gamma_k(N_{\rm a}z_{\rm ad} + N_{\rm d}z_{\rm da})(a_k^\dagger b_k^\dagger + a_k b_k), \end{aligned} \tag{8}$$

Now from the above Hamiltonian, if we consider the Heisenberg equations of motion for the magnon operators a_k and $b_k^\dagger$ (i.e. $i\hbar(da_k/dt) = [a_k, H_k]$) and assuming $\exp(-i\omega_{\rm m}t)$ time dependence for a_k and $b_k^\dagger$, we obtain the secular equation which directly yields the $\omega_{\rm m}$ dispersion, as follows (for $S_{\rm a} = S_{\rm d} = S$):

$$\hbar\omega_{\rm m} = g\mu_{\rm B}B_0 + \frac{J_{\rm ad}S(z_{\rm ad}N_{\rm a} + z_{\rm da}N_{\rm d})}{2}\left\{\left(\frac{1}{N_{\rm d}} - \frac{1}{N_{\rm a}}\right) \pm \left[\left(\frac{1}{N_{\rm d}^2} + \frac{1}{N_{\rm a}^2}\right) + \frac{2(1-2\gamma_k^2)}{N_{\rm a}N_{\rm d}}\right]^{1/2}\right\}, \tag{9}$$

which after re-grouping inside the $[]^{1/2}$ term, it becomes:

$$\hbar\omega_{\mathrm{m}} = g\mu_{\mathrm{B}}B_0 + \frac{J_{\mathrm{ad}}S(z_{\mathrm{ad}}N_{\mathrm{a}} + z_{\mathrm{da}}N_{\mathrm{d}})}{2N_{\mathrm{a}}N_{\mathrm{d}}}\left\{-\left(N_{\mathrm{d}} - N_{\mathrm{a}}\right) \pm \left[\left(N_{\mathrm{d}} - N_{\mathrm{a}}\right)^2 + 4(1-\gamma_k^2)N_{\mathrm{a}}N_{\mathrm{d}}\right]^{1/2}\right\}, \tag{10}$$

if we assume cubic symmetry for simplicity, and $ka << 1$ the above expression can be simplified to:

$$\hbar\omega_{\mathrm{m}} = g\mu_{\mathrm{B}}B_0 + \frac{J_{\mathrm{ad}}S(z_{\mathrm{ad}}N_{\mathrm{a}} + z_{\mathrm{da}}N_{\mathrm{d}})}{2}\left\{-\left(\frac{N_{\mathrm{d}} - N_{\mathrm{a}}}{N_{\mathrm{a}}N_{\mathrm{d}}}\right) \pm \left[\left(\frac{N_{\mathrm{d}} - N_{\mathrm{a}}}{N_{\mathrm{a}}N_{\mathrm{d}}}\right)^2 + \frac{4k^2a^2}{3N_{\mathrm{a}}N_{\mathrm{d}}}\right]^{1/2}\right\}, \tag{11}$$

the + and - solutions of the above magnon dispersion represent the acoustic and optical magnon branches respectively. For the problem we are now concerned, we only need to consider the acoustic dispersion branch (+ solution).

To consider the effect of the number of magnetic ions on each of the sites, we can use the expressions: $N_{\mathrm{a}} = \lambda N$ and $N_{\mathrm{d}} = \mu N$, where N refer to the number of Fe^{3+} ions per unit volume and λ (μ) correspond to the ion density in the octahedral (tetrahedral) sites. Introducing this notation into the dispersion described previously, we obtain:

$$\hbar\omega_{\mathrm{m}} = g\mu_{\mathrm{B}}B_0 + \frac{J_{\mathrm{ad}}S(z_{\mathrm{ad}}\lambda + z_{\mathrm{da}}\mu)}{2}\left\{-\left(\frac{\mu - \lambda}{\lambda\mu}\right) \pm \left[\left(\frac{\mu - \lambda}{\lambda\mu}\right)^2 + \frac{4k^2a^2}{3\lambda\mu}\right]^{1/2}\right\}, \tag{12}$$

this expression is similar to Eq. 2 of the main text with the external magnetic field $B_0 = \mu_0 H$ and the gyromagnetic ratio $\gamma = g\mu_{\mathrm{B}}/\hbar$. The effect of non-magnetic ion substitution can be accounted for by introducing the following relations[21]:

$$z_{\mathrm{da}} = 4\left(\frac{2-x}{2}\right) \qquad z_{\mathrm{ad}} = 6\left(\frac{3-y}{3}\right) \tag{13}$$

$$\mu = 0.6\left(\frac{3-y}{3}\right) \qquad \lambda = 0.4\left(\frac{2-x}{2}\right), \tag{14}$$

where x and y are the concentration of non-magnetic ion substitutions per unit formula in octahedral (a) and tetrahedral (d) sites, respectively.

SUPPLEMENTARY NOTE 5: CASE OF YIG ($x = y = 0$)

To test the validity of our model, we first consider the case of $Y_3Fe_5O_{12}$ (YIG) with $x = y = 0$. Supplementary Figure 4a shows the SSE measurement at 300 K for a YIG film, and Supplementary Figure 4b shows the data blown-up of the SSE voltage in the region where magnon-polaron SSE peak is observed for YIG. Now if we consider the magnon dispersion previously obtained in Eq. 12, together with the expressions in Eq. 13, Eq. 14, and using $a = 3.46 \times 10^{-10}$ m (nearest-neighbour distance between a-d sites in YIG)[22]. We can estimate the value of the inter-site exchange parameter by considering the tangential condition between the magnon and TA phonon dispersion, obtaining $|J_{\rm ad}| = (4.3 \pm 0.2) \times 10^{-22}$ J, in close agreement to the value recently obtained by Shamoto et al. using neutron scattering measurements[17]: $J_{\rm ad} = 4.65 \times 10^{-22}$ J.

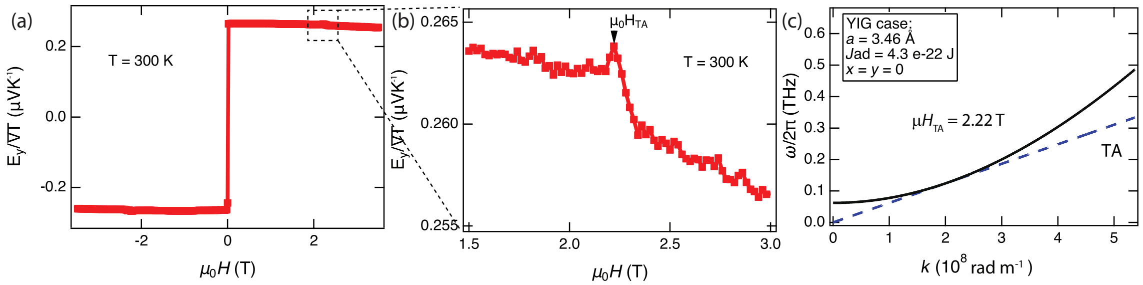

Supplementary Figure 4. **a** Magnetic field dependence of the SSE voltage in YIG, the detail of the magnon-polaron peak due to hybridization of magnons with TA phonons is shown in **b**. **c** Comparison of the magnon and phonon dispersions at the magnetic field $H_{\rm TA}$ in **b**, inset shows the parameters used for the determination of magnon dispersion. The value of $c_{\rm TA} = 3.9 \times 10^3$ ms^{-1} is the same as that used by Kikkawa et al.[4]

SUPPLEMENTARY NOTE 6: EFFECT OF NON-MAGNETIC ION SUBSTITUTION ON THE QUADRATIC (k^2) COMPONENT OF THE MAGNON DISPERSION

The magnon-polaron peaks in SSE have been explained by the larger magnon-phonon hybridization over k-space, when the condition for tangential touching between magnon and phonon dispersions is met. Therefore, we want to evaluate the degree of magnon-phonon overlap over k-space as a function of magnetic compensation (non-magnetic substitutions) at the touching condition. The touch condition is met when $\omega_{\rm m} = \omega_{\rm p}$ and $\frac{\partial \omega_{\rm m}}{\partial k} = \frac{\partial \omega_{\rm p}}{\partial k}$, with $\omega_{\rm m}$ and $\omega_{\rm p}$ being the frequency of magnon and phonon dispersions, respectively.

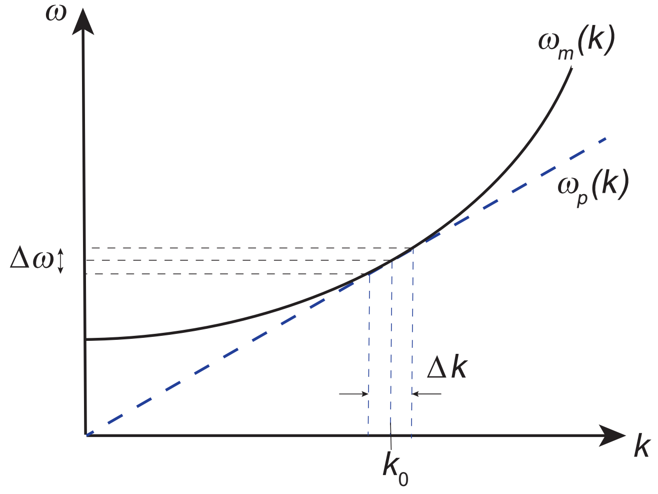

Supplementary Figure 5. Parameters of the power expansion of the magnon dispersion around the touching point (k_0) condition with $\Delta\omega = \omega_{\rm m}(k-k_0) - \omega_{\rm m}(k_0)$ and $\Delta k = k - k_0$.

In order to evaluate the degree of overlap, we can expand the magnon dispersion as a k power series around the touching point k_0 (see Fig. 5), given by the formula:

$$\omega_{\rm m}(k-k_0) = \omega_{\rm m}(k_0) + \left.\frac{\partial\omega_{\rm m}}{\partial k}\right|_{k=k_0}(k-k_0) + \left.\frac{\partial^2\omega_{\rm m}}{\partial k^2}\right|_{k=k_0}(k-k_0)^2 + ... \tag{15}$$

At the tangential touching point k_0, both magnon and phonon dispersions have the same velocity (i.e. their first derivatives are the same: $\frac{\partial\omega_{\rm m}}{\partial k}\Big|_{k=k_0} = \frac{\partial\omega_{\rm p}}{\partial k}\Big|_{k=k_0} = c_{\rm p}$, where $c_{\rm p}$ is the phonon velocity). Therefore, in order to evaluate the degree of magnon-phonon overlap in k-space, we need to consider the quadratic component, which represents the deviation from the linear behaviour at the touching point and it should decrease in order to increase the magnon-phonon overlap.

Before evaluating the power expansion series, let us re-express the magnon dispersion obtained in Eq. 12 as:

$$\omega_{\rm m} = \omega_z + A\left[-B \pm \left(B^2 + Ck^2\right)^{1/2}\right], \tag{16}$$

where we have introduced the following notation: $\omega_z = \gamma\mu_0 H$ is the Zeeman gap induced by the external magnetic field, $A = \frac{J_{\rm ad}S(z_{\rm da}\mu + z_{\rm ad}\lambda)}{2\hbar}$, $B = \frac{\mu-\lambda}{\mu\lambda}$, $C = \frac{4a^2}{3\lambda\mu}$ and $D = \left(\frac{A}{c_{\rm p}}\right)^2 C$. From the tangential touching condition ($\frac{\partial\omega_{\rm m}}{\partial k} = \frac{\partial\omega_{\rm p}}{\partial k} = c_{\rm p}$), we can obtain the value of k_0 at the touching point:

$$k_0 = \pm\frac{B}{\sqrt{\left(\frac{AC}{c_{\rm p}}\right)^2 - C}}. \tag{17}$$

Using the above expression we can evaluate the magnitude of the quadratic component at k_0, which should be minimized in order to obtain the largest overlap over k-space. This can be obtained from the second order component of the Taylor expansion around k_0, which gives the expression for the quadratic term:

$$\left.\frac{\partial^2 \omega_{\mathrm{m}}}{\partial k^2}\right|_{k=k_0} = \frac{AC}{B}\left(\frac{D-1}{D}\right)^{1/2}\left(1+\frac{1}{D}\right), \tag{18}$$

with the parameters A, B, C, D as defined previously. Now, we can obtain the dependence of the quadratic component as a function of the tetrahedral site substitution, y (see Supplementary Figure 6).

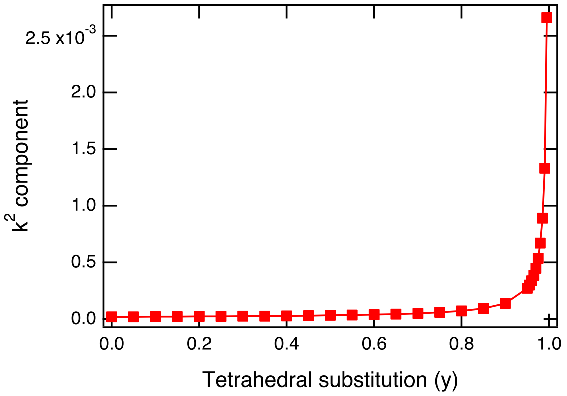

Supplementary Figure 6. Estimation of the magnitude of the quadratic component of the magnon dispersion as a function of the tetrahedral site substitution ($0 \leq y < 1$).

We can see that, counter-intuitively, the k^2 component gradually increases with the tetrahedral site substitution y (proportional to Ga doping). Therefore the relatively larger magnitude of the magnon-polaron peaks in BiGa:LuIG (compared to YIG) cannot be explained by an increased overlap over k-space. This is clearly illustrated in Supplementary Figure 7, showing the comparison of the magnon and phonon dispersions at the touching condition for BiGa:LuIG and YIG, where we can see that the overlap over k-space is actually larger in the case of YIG.

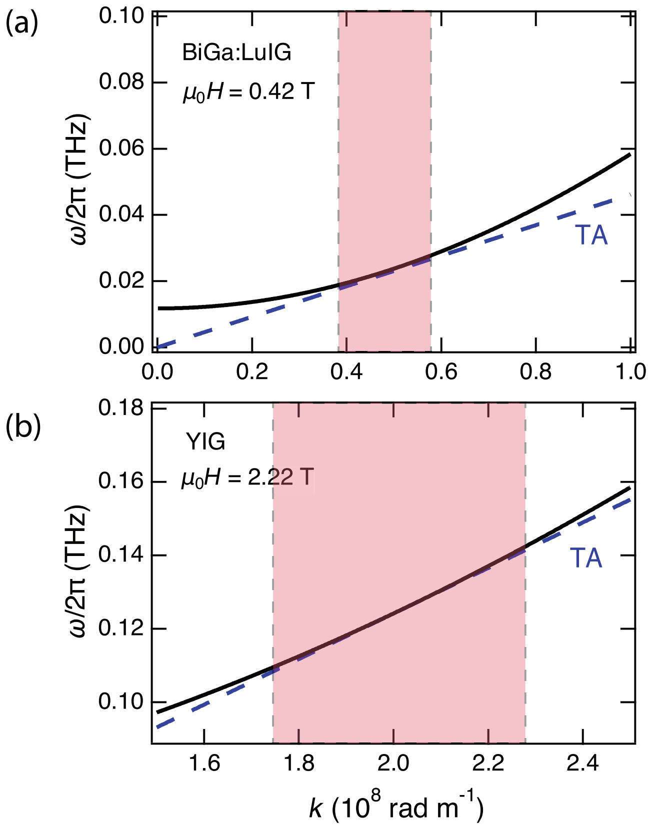

Supplementary Figure 7. Comparison of the magnon-phonon dispersions at magnetic fields H_{TA} for the touching condition between the magnon and TA phonon dispersions in **a** BiGa:LuIG ($J_{\mathrm{ad}} = 4 \times 10^{-22}$ J, $x = 0.101$, $y = 0.909$ and $a = 0.346$ nm) and **b** YIG ($J_{\mathrm{ad}} = 4.3 \times 10^{-22}$ J, $x = y = 0$ and $a = 0.346$ nm). Red shadow area represents the overlap region. Note that the plotted range in both frequency ($\omega/2\pi$) and wavenumber (k) axes is the same for both figures.

SUPPLEMENTARY REFERENCES

* Corresponding author: ramosr@imr.tohoku.ac.jp

[1] Z Qiu, D Hou, K Uchida, and E Saitoh, "Influence of interface condition on spin-Seebeck effects," J. Phys. D: Appl. Phys. **48**, 164013 (2015).

[2] P. Hansen, C.-P. Klages, J. Schuldt, and K. Witter, "Magnetic and magneto-optical properties of bismuth-substituted lutetium iron garnet films," Phys. Rev. B **31**, 5858 (1985).

[3] T. Kikkawa, K. Uchida, S. Daimon, Z. Qiu, Y. Shiomi, and E. Saitoh, "Critical suppression of spin Seebeck effect by magnetic fields," Phys. Rev. B **92**, 064413 (2015).

[4] T. Kikkawa, K. Shen, B. Flebus, R. A. Duine, K. Uchida, Z. Qiu, G. E. W. Bauer, and E. Saitoh, "Magnon Polarons in the Spin Seebeck Effect," Phys. Rev. Lett. **117**, 207203 (2016).

[5] A. Aqeel, I. J. Vera-Marun, B. J. van Wees, and T. T. M. Palstra, "Surface sensitivity of the spin Seebeck effect," J. Appl. Phys. **116**, 153705 (2014).

[6] P. Hansen, P. Rschmann, and W. Tolksdorf, "Saturation magnetization of gallium-substituted yttrium iron garnet," J. Appl. Phys. **45**, 2728 (1974).

[7] S. Chikazumi, *Physics of Ferromagnetism* (Oxford University Press, New York, 1997).

[8] C. L. Jermain, H. Paik, S. V. Aradhya, R. A. Buhrman, D. G. Schlom, and D. C. Ralph, "Low-damping sub-10-nm thin films of lutetium iron garnet grown by molecular-beam epitaxy," Appl. Phys. Lett. **109**, 192408 (2016).

[9] K. Uchida, Z. Qiu, T. Kikkawa, R. Iguchi, and E. Saitoh, "Spin Hall magnetoresistance at high temperatures," Appl. Phys. Lett. **106**, 052405 (2015).

[10] K. Uchida, J. Ohe, T. Kikkawa, S. Daimon, D. Hou, Z. Qiu, and E. Saitoh, "Intrinsic surface magnetic anisotropy in $Y_3Fe_5O_{12}$ as the origin of low-magnetic-field behavior of the spin Seebeck effect," Phys. Rev. B **92**, 014415 (2015).

[11] P.-H. Wu and S.-Y. Huang, "Noncollinear magnetization between surface and bulk $Y_3Fe_5O_{12}$," Phys. Rev. B **94**, 024405 (2016).

[12] V. Kalappattil, R. Das, M.-H. Phan, and H. Srikanth, "Roles of bulk and surface magnetic anisotropy on the longitudinal spin Seebeck effect of Pt/YIG," Scientific Reports **7**, 13316 (2017).

[13] Dmytro A. Bozhko, Alexander A. Serga, Peter Clausen, Vitaliy I. Vasyuchka, Frank Heussner, Gennadii A. Melkov, Anna Pomyalov, Victor S. L'vov, and Burkard Hillebrands, "Supercurrent

in a room-temperature Bose–Einstein magnon condensate," Nat. Phys. **12**, 1057 – 1062 (2016).

[14] A. A. Serga, V. S. Tiberkevich, C. W. Sandweg, V. I. Vasyuchka, D. A. Bozhko, A. V. Chumak, T. Neumann, B. Obry, G. A. Melkov, A. N. Slavin, and B. Hillebrands, "Bose–Einstein condensation in an ultra-hot gas of pumped magnons," Nat. Comm. **5**, 3452 (2014).

[15] S. M. Rezende, R. L. Rodríguez-Suárez, R. O. Cunha, A. R. Rodrigues, F. L. A. Machado, G. A. Fonseca Guerra, J. C. Lopez Ortiz, and A. Azevedo, "Magnon spin-current theory for the longitudinal spin-Seebeck effect," Phys. Rev. B **89**, 14416 (2014).

[16] J. Barker and G. E. W. Bauer, "Thermal spin dynamics of yttrium iron garnet," Phys. Rev. Lett. **117**, 217201 (2016).

[17] S. Shamoto, T. U. Ito, H. Onishi, H. Yamauchi, Y. Inamura, M. Matsuura, M. Akatsu, K. Kodama, A. Nakao, T. Moyoshi, K. Munakata, T. Ohhara, M. Nakamura, S. Ohira-Kawamura, Y. Nemoto, and K. Shibata, "Neutron scattering study of yttrium iron garnet," Phys. Rev. B **97**, 054429 (2018).

[18] Stancil D. D. and Prabhakar A., *Spin Waves: Theory and Applications* (Springer, New York, 2009).

[19] M. Sparks, *Ferromagnetic-relaxation theory* (McGraw-Hill, New York, 1964).

[20] W. Nolting and A. Ramakanth, *Quantum theory of magnetism* (Springer-Verlag, Berlin Heidelberg, 2009).

[21] N. Miura, I. Oguro, and S. Chikazumi, "Computer simulation of temperature and field dependences of sublattice magnetizations and spin-flip transition in gallium-substituted yttrium iron garnet," J. Phys. Soc. Jpn. **45**, 1534–1541 (1978).

[22] S. Geller and M.A. Gilleo, "The crystal structure and ferrimagnetism of yttrium-iron garnet, $Y_3Fe_2(FeO_4)_3$," J. Phys. Chem. Sol. **3**, 30 – 36 (1957).
